# An integrated theory based-educational intervention to change intention to have a child: study protocol of a cluster randomized controlled trial

**DOI:** 10.1186/s12978-024-01760-x

**Published:** 2024-02-28

**Authors:** Maryam Moridi, Maryam Damghanian, Sedigheh Keshaverz

**Affiliations:** 1 Reproductive Health and Midwifery Department, School of Nursing and Midwifery, Tehran University of Medical Sciences, Tehran, Iran; 2 Department of Reproductive Health, School of Nursing and Midwifery, Tehran University of Medical Sciences, Tehran, Iran; 3 Nursing and Midwifery Care Research Center, School of Nursing and Midwifery, Tehran University of Medical Sciences, Tehran, Iran

**Keywords:** Childbearing, Stages of change model, Theory of planned behavior, Training couple

## Abstract

**Background:**

In high- and low-income countries, declining birth rates have become a global concern. Couples do not have enough information about the complications of delaying and reducing childbearing and this leads them to make inappropriate decisions. Therefore, this study aims to evaluate whether an educational program based on integrating the Theory of Planned Behavior (TPB) and the Trans-Theoretical Model (TTM) affects child-free couples’ intention to have children and minimizes the consequences of this decline.

**Methods:**

Thirty couples (intervention, n = 15; control, n = 15) will be enrolled in this cluster randomized controlled trial. After collecting baseline data and separating participants in the pre-contemplation and contemplation stages based on the TTM, the samples were randomly assigned to the intervention and control groups. The intervention group will receive 60-min training based on TPB components for 4 weeks. The first follow-up assessment was performed immediately after the intervention and the final assessment 6 months later. For all 3 time assessments, three questionnaires will be used: The knowledge questionnaire, the TTM, and the TPB questionnaire. The most important consequences are changes in knowledge, attitudes, subjective norms, perceived behavioral control, and stages of intentions to have children.

**Discussion:**

Decision-makers will use the results of this study as a basis to design appropriate, transparent, and useful policies and interventions to improve or stop the decline of the fertility rate at the national level. Also, this study will help young couples who wish to have a child in their lifetime by providing relevant information so that they do not miss this opportunity and face the consequences of delaying having a child.

*Trial registration* This study was approved by the Iranian Registry of Clinical Trials (IRCT), Number: IRCT20220618055210N2, Date of registration: 2023-10-03

## Background

Childbearing is one of the main factors of population dynamics and plays an important role in the change in population size and structure [1, 2]. This phenomenon is a multidimensional concept with familial, social, economic, cultural, political, and religious implications [3]. One of the significant concerns, which began at the end of the nineteenth century, is the decreasing fertility rate, which has been felt throughout the world [4–6].

Iran as a developing country, has experienced a sharp decline in the fertility rate, and during the last three decades [7], the total fertility rate (TFR), has fallen from 6.3 in 1986 to 1.62 in 2018[8]. It is estimated that Iran’s population growth will reach 1% by 2025 [9] and will fall to less than 1% by 2031 [10]. This is while in the religious and cultural context of Iran, having children is a virtue and one of the main motivations for getting married and starting a family, and couples’ families expect couples to have their first child soon after marriage [11].

One of the main causes of the fertility rate decline is changing fertility patterns and the increasing average age at marriage [12]. Delayed childbearing increases the risk of unintended infertility and limits the number of children in each family [13]. However, studies have shown that couples believe that their reproductive behavior is completely under their control and are often unaware of the consequences of late conception and increasing age at first pregnancy [2, 14]. Increasing parental age is associated with increased infertility and fertility risks such as miscarriage; ectopic pregnancy; multiple pregnancies; low birth weight; gestational hypertension and diabetes; maternal mortality; stillbirth; premature births; down syndrome; and birth defects including congenital heart disease, cleft palate, esophageal atresia, schizophrenia; poor neurodevelopment; and the likelihood of childhood cancer [13, 15, 16].

Unfortunately, Iranian women are largely unaware of the potential side effects of delaying childbirth, and the issue appears to be ignored by the Iranian health system [17]. This lack of knowledge inadvertently exposes them to infertility and fertility disorders, thereby increasing the need for Assisted Reproductive Technology (ART) [18]. Despite the considerable advances in ART methods as well as the public imagination and expectations regarding the successfulness of these methods, they still cannot produce conclusive childbearing or compensate for the decline in fertility as women age [19, 20]. Even in developed countries, neither women nor men fully understand the effect of age on fertility and this lack of knowledge is more common among men who are unaware of the negative effects of age, smoking, consuming alcoholic beverages, and obesity on fertility [21, 22]. Currently, many public policies are being considered to reduce such effects [23]. Consequently, in 2013, Iran enacted the Family Support Law to address these issues. However, proper implementation requires a change in the attitude of couples [24].

Since behavioral intention is a key factor in the performance of that behavior [2], it can be concluded that one effective way to change people's behavioral intention is to educate them individually or in groups to increase knowledge about fertility and change their intention [19]. Based on research, the use of theoretical models and frameworks in the design of such educational activities can increase their effectiveness [25]. Recently, the TPB has been used as a framework to address this issue, as its effectiveness in childbearing has been demonstrated in several studies [8] and it can help to understand how couples make decisions about having a child by establishing a link between attitudes and behaviors [26, 27]. According to the TPB, three determining factors influence behavioral intention: 1) the attitude, 2) subjective norms, and 3) perceived behavioral control [25, 28].

In European countries, the intention to have a first child is influenced by attitudes towards having children and subjective norms [2]. To encourage couples to decide to have children, it is necessary to find a way to alter their attitudes and subjective norms and the three components of TPB can help achieve this goal [26]. The TPB provides important information about what influences behavioral intentions but does not adequately explain the extent and manner of desired behavior change [29]. For this purpose, the TTM model can well describe the process of change or how people move toward the desired change [30, 31]. Knowing where a person is in the stage of change is useful for designing appropriate interventions that encourage the person to make the desired changes [32]. The TTM model includes 5 constructs: stages of change, the process of change, levels of change, self-efficacy, and decision balance; the most popular of these is "stages of change", which can determine how health behaviors change [33]. This dimension has 6 steps: Pre-contemplation, Contemplation, Preparation, Action, Maintenance, and Termination [34]. The effectiveness of this theory in changing behavior has been confirmed in several fields, and an intervention study in Iran also used this theory to change childbearing behavior [26]. However, limited research has used a combination of these two models to change behavior, and based on a review, a combination of these two theories has been used to change healthy behavior in exercise, cancer screening, diet, and smoking behavior [34].

The average age of marriage in Iran is 27 [35]. About 30% of women experience their first pregnancy within 5 years of marriage, and about 4% remain childless after 10 years of marriage [17]. On the other hand, female infertility starts to increase from the age of 30, and by the age of 40, 1 in 6 women are no longer able to conceive, and after age 40, more than half of women lose their ability to conceive [21]. Therefore, the present study intends to provide them with the right information before facing the decline in fertility. The assumption is that informing couples can help them make appropriate decisions about having children so that even fewer couples will suffer mental and physical disability from infertility and its high medical costs in the future. On the other hand, it is necessary to minimize the consequences of an aging society to an appropriate level. In this study, the plan is to use the TPB theory to train people and measure people's behavior changes after an intervention based on the TTM model, because this model is very effective in accurately measuring the stages of change and the amount of change.

**Hypothesis**

An educational intervention based on the integration of TTM and TPB on the intention to have a child in childless couples leads to changes in knowledge, attitude, subjective norms, perceived behavioral control, and intention to have a child, which in turn changes behavior, which can lead to strategies to solve problems related to the aging population.

## Methods/design

### Study aims

#### The main aim

The main objective of this study is to evaluate the effectiveness of an integrated theory-based educational intervention on the intentions of childless couples to have a child.

#### Secondary aims

1. Evaluate changes in intentional stages.
2. Assess the impact of the training program on factors influencing behavior (attitude, subjective norms, perceived behavioral control, and behavioral intention).
3. To investigate the relationship between demographic characteristics of couples and the intention to have children.

### Trial design

**Fig. 1:**
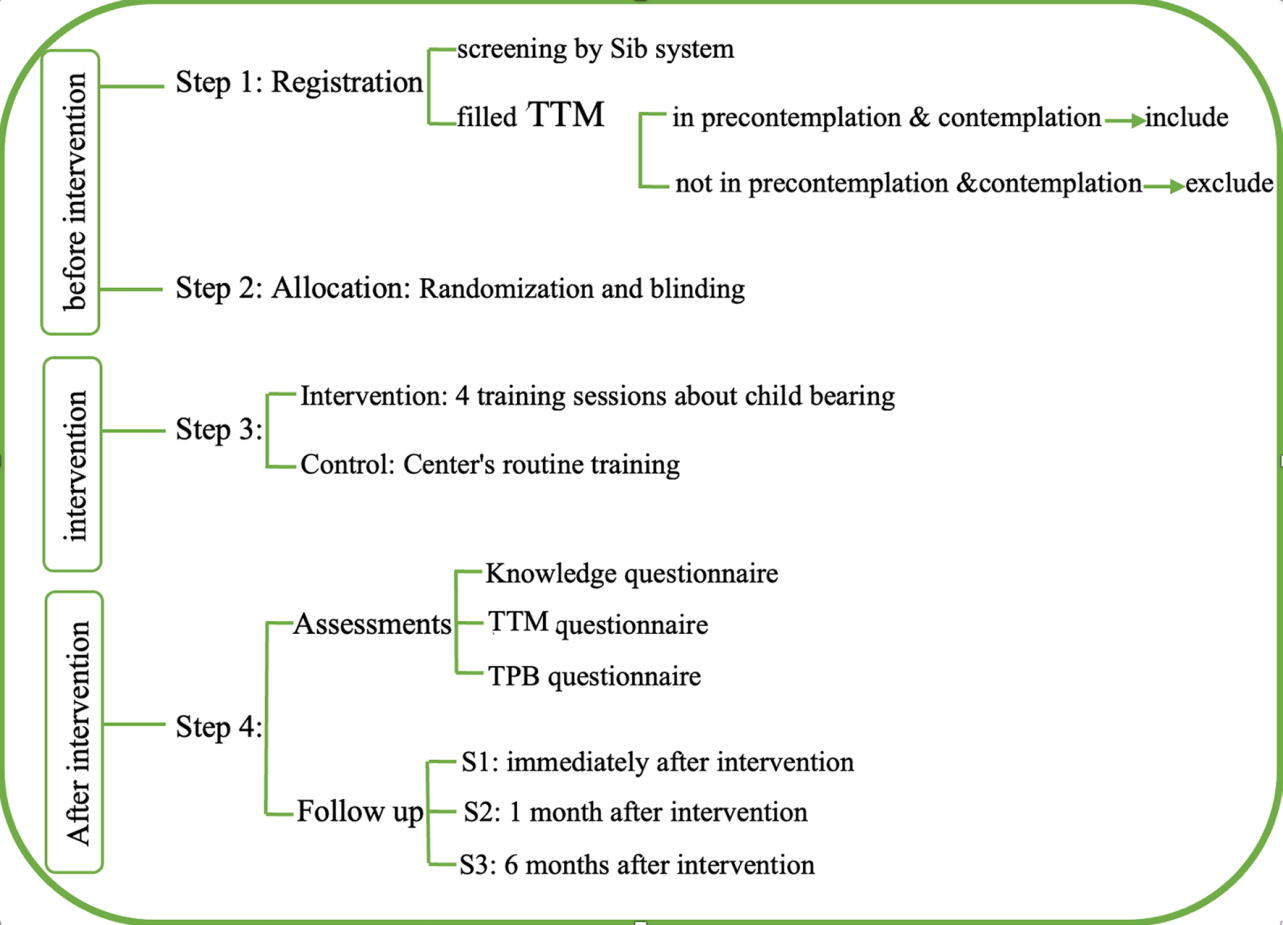
Research process

This study is a parallel group clinical trial. The TTM and TPB are used for training and measuring outcomes. The participants are childless couples who have been married for at least 2 years and are in the pre-contemplation and contemplation stages of having children. A diagram of the research process is shown in Fig. 1. After the intervention, their attitudes, subjective norms, and perceived level of control over childbearing behavior will be compared with before the intervention, to determine whether the interventions lead couples out of the pre-contemplation stage to later stages or not. The duration of this study is approximately 2 years from the beginning of the training to the end of the follow-up (6 months after the intervention). This study was approved by the Ethics Committee (Ethics Code: IR.TUMS.FNM.REC.1402.013) of Tehran Medical University. Informed consent is completed by all participants after an explanation of the objectives of the study, the research process, and the follow-up period.

### Setting and participants

The intervention will take place in Tehran, the capital of Iran. Samples from the control and intervention groups will be selected from the Primary Health Center (PHC). Participants will be 30 couples who have been married for at least 2 years or more and have not been diagnosed with infertility. The couple's infertility will be determined based on their previous medical records available at medical centers.

### Inclusion criteria

Aged from 18 to 40 years old for women and up to 45 years old for men, want to participate in research, being in the pre-contemplation or contemplation stage, married for 2 years or more, no contraindications to pregnancy, have no history of infertility, use of contraceptives, have no mental disability that interferes with learning.

### Exclusion criteria

Unwillingness to participate in the study, not attending more than one session, diagnosed infertility, or any chronic disease contraindicated for pregnancy.

### Interventions

After receiving the ethical code from the Research Center of Tehran University of Medical Sciences, the study will begin. The educational content of this study will be prepared under the supervision of specialized professors of the university and the researcher will acquire the necessary knowledge and skills to carry out the educational program under the supervision of professors. In preparing this content, we will try to include the following: Complications due to reduced population growth, factors affecting the reduced ability to have children, effects of reduced population growth on family and society, complications of pregnancy at older ages, effects of father's age, benefits of having children, economic effects of demographic decline. The choice of the content is based on: the educational needs of the couples reflected in the studies, the cultural problems of the society, and the subjects mentioned by the Ministry of Health of Iran. After the teaching materials are prepared, their contents are scientifically evaluated by the Tehran University of Medicine professors, and after their approval, the intervention and training process will begin. Teaching classes will be conducted in 4 sessions and each session will last at least 60 min with the presence of couples (Table 1).

**Table 1:** The content of the educational sessions based on the theory of planned behavior

session	Main purpose	Educational materials	Educational methods and time schedule
1	Improving couples’ knowledge and attitudes toward	PowerPoint Booklet (including contents of all sessions) Pamphlet of the first session	1) Introducing presenter and participants and explaining study aims (10 min) 2) Giving information on effect of advanced ages on fertility capacity of couples and the pregnancy’s complications (40 min) 3) Question and answer (10 min)
2	Addressing the role of subjective norms in childbearing	PowerPoint Pamphlet of the second session	1) Reviewing previous session (5 min) 2) Giving information related to Islamic beliefs on childbearing and the importance of having a child on the religious and social perspectives of Iran (15 min) 3) Presenter will use the psychodrama method. In this method two female volunteer who have at least one child role‐play problems and release their intense emotions in regard to the child, then the presenter will guide their subjective norms (37) (30 min) 4) Question and answer (10 min)
3	Addressing the role of perceived behavioral control in childbearing	PowerPoint Pamphlet of the third session Video	1) Review of previous session (5 min) 2) Giving information on requirements and cares for becoming pregnant, prenatal cares, and care of newborn (30 min) 3) Presenting a short video which shows how a baby develops (5 min) Showing how to provide care during pregnancy and for newborn (10 min) 4) Showing videos of care of newborn (10 min) 5) Question and answer (10 min)
4	Addressing the behavioral intention and explaining how childbearing will change couple’s life	PowerPoint Pamphlet of the fourth session Video	1) Reviewing previous session (5 min) 2) Giving information on advantages of having a child from cultural, religious and social perspectives (10 min) 3) Showing videos of successful childbearing expression (10 min) 4) Question and answer (10 min)

Participants who meet the inclusion criteria will first complete a childbearing intentions questionnaire, which will be designed based on the stages of the change model and the samples who are in the pre-contemplation and contemplation stage will be included in the study. After this step, by accessing the Sib system “a national system that records health information of individuals”, couples who have been married for 2 years without children and meet the inclusion criteria will be selected. Then, 60 samples (30 pairs) will be selected through a random number table and will be randomly divided into 2 groups: control and intervention.

Teaching methods will include conferences, discussions, Q&A, and document distribution. During the classes, the researcher does not impose personal views and attitudes on the couple. It is explained to them that all decisions about having children are based entirely on their opinions and considerations and there is no coercion. They must also fill in the questionnaires without concern and honesty because their answers do not affect the quantity and quality of the treatment, and whenever they do not want to participate in the study, they can refuse to continue.

### Outcomes

#### Primary outcome

As mentioned, the most important thing in a couple's decision to have children is the intention to do so and a positive attitude towards it, so:

- Change the behavior of couples to the operation and maintenance phase according to the TTM after the procedure
- Change to a positive attitude toward having a baby.

#### Secondary outcomes

- Increase awareness among couples about complications related to delaying childbirth
- Increases the couple's sense of reproductive control
- Improve the capacity of couples to cope with the spiritual standards of society
- Increase couples' preparedness to make informed fertility decisions.

### Data collection

A questionnaire designed by researchers will be used to assess the impact of this intervention on people's attitudes and subjective norms. This questionnaire is administered to assess the three components of attitude, subjective norms, and perceived behavioral control and to determine how the change process is carried out in four stages: before the intervention (S0), immediately after the intervention (S1), 1 month after the intervention (S2) and 6 months after the procedure (S3).

The questions prepared for this study will be designed based on: Research objectives, two mentioned models, and teaching content. Once designed by the researcher, it will be sent to specialist teachers and their validity and reliability will be assessed. Questions in the S0 phase are filled in person at the clinic, but to allow people to better receive questions and answers without the need to visit the clinic, the questions asked in the questionnaire will be sent online in the later stages. This Iranian online system is called Porsline, and at an agreed time, a link to the survey is sent to participants, and the researcher monitors their responses by phone.

### The study instruments

#### 1Demographic information questionnaire

This questionnaire will include the couple's demographic information: age, education level, occupation, income, age at marriage, and duration of marriage. Additionally, information about a woman's fertility will include abortion, age at first menstruation, menstrual cycle regularity, and possible prevention methods. This questionnaire will only be completed in person at the beginning of the study.

#### Level of knowledge

The questions in this section are based on the material taught in the training sessions and are designed to investigate changes in the level of awareness of participants, as increasing the awareness of couples can help them make better decisions in the field of reproduction [29].

#### Trans-theoretical model questionnaire (TTM)

At the beginning of the study, to find out whether the person meets the criteria to enter the study or not, the participant must fill out a questionnaire about the stages of change, and whether they are in the pre-contemplation or contemplation stage, it is included in the study. This questionnaire is designed based on the stages of the change model and includes 7 questions to determine which stage the person is in. After the intervention, each participant's level of change toward the next steps in behavioral intentions will be measured using this questionnaire according to specific steps.

#### Theory of planning behavior questionnaire (TPB)

The next questionnaire developed by the researchers will be based on the theory of planned behavior. According to this theory, human behavior is directly determined by three main factors: attitudes, subjective norms, and perceived behavioral control [27]. Therefore, the designed questionnaire will also have three parts. 15 questions will measure individual attitudes, 10 questions will measure subjective norms and 25 questions will measure perceived behavioral control of the couples. Due to the influence of different environmental factors on the "Perceived behavioral control" factor, questions related to economic issues (6 questions), employment and work-related factors (5 questions), educational factors (2 questions), social factors (6 questions), questions about physical factors (3 questions) and mental factors (3 questions) are designed separately.

### Validity and reliability

In this study, three questions need to be tested for validity and reliability:

Knowledge level, TTM, and TPB. The reliability of the questions will be determined by its internal consistency (Cronbach's alpha) and the content validity of the questions will be assessed using both qualitative and quantitative methods. In the qualitative method, 10 experts evaluate the questionnaire, including faculty members of the Department of Midwifery and Reproductive Health of Tehran University of Medical Sciences, and after receiving their opinions, the proposed changes will be applied. The Content Validity Index (CVI) and Content Validity Ratio (CVR) are measured quantitatively. For face validity, we ask 10 couples to give comments on the clarity of the survey, and based on their opinions, basic explanations are given, if the survey is unclear from the couple's point of view, the necessary corrections will be performed.

### Sample size

To provide a power of 95% with a 99% confidence interval using the following formula and considering the mean and standard deviation of intention to have based on the findings of a similar study by Moghaddam et al. [36]. The sample size was estimated at 12, and then considering 20% attrition, we plan to recruit 15 couples per group and a total of 30 couples study participants.

$$n=\frac{{( {z}_{1-\frac{\alpha }{2}}+ {z}_{1-\beta } )}^{2} ({s}_{1}^{2}+ {s}_{2}^{2})}{{({\mu }_{1}- {\mu }_{2})}^{2}}=\frac{2.1664 \times 14.82}{2.6896}=\frac{32.106048}{2.6896}=11.93 \cong 12$$

### Randomization

After checking the participants' basic information regarding the inclusion criteria, a list will be prepared, and then, to randomly divide them into two intervention and control groups, samples will be allocated into two groups based on the Random Number Table. Because interventions aim to educate people, participants cannot be blinded. The people targeted by the intervention are therefore aware of the group to which they belong.

### Blinding

Additionally, there was no possibility of blinding the researcher due to the organization of the training sessions and the conduct of the interventions. However, medical center staff will be blinded. Blinding is also done when dividing the participants into two control and intervention groups and during the statistical analysis of the data. Training sessions for the intervention group will take place on days when people in the control group are not present at the clinic so that information is not randomly delivered to them and they do not communicate with each other. This issue will be the reason for the long process (about 2 years) of this research.

### Statistical analysis

The analytical method used in this study will be quantitative. The information recorded in the questionnaire will be entered into the SPSS program at each stage and data analysis will be conducted after the research process is completed. Categorical variables will be reported based on frequency, and quantitative variables will be reported as mean (SD). The ANOVA test will be used for quantitative variables that have a normal distribution, and the Friedman test will be used for quantitative variables that do not have a normal distribution. The effectiveness of the intervention will be determined using an ANOVA test and the impact of demographic variables on factors measured using a logistic regression model. Statistical analyses will be performed using IBM-SPSS 22. For all analyses, P values  < 0.05 are considered statistically significant.

### Ethics approval and ethical considerations

1. Receive the Code of Ethics and Letter of Recommendation from the Medical Ethics Council of the Faculty of Nursing and Midwifery (Ethics Code: IR.TUMS.FNM.REC.1402.013).
2. Get permission to enter the medical center where the study is taking place.
3. Respect ethical principles when conducting research.
4. Present the research sample to the researcher and explain the objectives.
5. Emphasis is placed on voluntary participation and obtaining informed consent.
6. Ensure confidentiality of information of all participants.
7. Announce accurate and realistic results.

## Discussion

Considering the global circumstances and the situation of our country regarding population decline and its consequences, it is very important to implement a properly designed educational program to improve awareness, attitude, and childbearing behavior change. Since Iran is a Muslim country, having a child is considered part of the culture and beliefs of families [19]. In such a context, we expect a favorable result from interventions in this field and we can help couples make timely decisions about having children. The hypothesis is that, due to a lack of awareness of the consequences of delaying pregnancy, many couples unwittingly experience complications such as infertility and its treatment costs, pregnancy complications, and age costs, and ultimately having fewer children than desired. We hope that decision-makers will use the results of this study as a basis to design appropriate, transparent, and useful policies and solutions to provide quality services and an appropriate culture to increase or prevent fertility decline at the national level. In addition, if the intervention is effective, the findings can be part of reproductive health counseling for couples in the future and can be combined with premarital counseling for couples.

## Data Availability

Not applicable, as this is a protocol manuscript.

## Abbreviations

TPB: Theory of planned behavior
TTM: The Transtheoretical Model
TFR: Total fertility rate
ART: Assisted Reproductive Technology
LBW: Low birth weight
EP: Ectopic pregnancy
